# Effect of environmental variables and *kdr* resistance genotype on survival probability and infection rates in *Anopheles gambiae* (*s.s.*)

**DOI:** 10.1186/s13071-018-3150-8

**Published:** 2018-10-26

**Authors:** Mojca Kristan, Tarekegn A. Abeku, Jo Lines

**Affiliations:** 1 Department of Disease Control, London School of Hygiene & Tropical Medicine, London, UK; 2 Malaria Consortium, London, UK

**Keywords:** Malaria, *Anopheles gambiae*, Insecticide resistance, Pyrethroids, *Plasmodium falciparum*, Oocyst, Sporogony, Environmental factors, Temperature

## Abstract

**Background:**

Environmental factors, especially ambient temperature and relative humidity affect both mosquitoes and malaria parasites. The early part of sporogony is most sensitive and is affected by high temperatures and temperature fluctuation immediately following ingestion of an infectious blood meal. The aim of this study was to explore whether environmental variables such as temperature, together with the presence of the *kdr* insecticide resistance mutations, have an impact on survival probability and infection rates in wild *Anopheles gambiae* (*s.s.*) exposed and unexposed to a pyrethroid insecticide.

**Methods:**

*Anopheles gambiae* (*s.s.*) were collected as larvae, reared to adults, and fed on blood samples from 42 *Plasmodium falciparum*-infected local patients at a health facility in mid-western Uganda, then exposed either to nets treated with sub-lethal doses of deltamethrin or to untreated nets. After seven days, surviving mosquitoes were dissected and their midguts examined for oocysts. Prevalence (proportion infected) and intensity of infection (number of oocysts per infected mosquito) were recorded for each group. Mosquito mortality was recorded daily. Temperature and humidity were recorded every 30 minutes throughout the experiments.

**Results:**

Our findings indicate that apart from the effect of deltamethrin exposure, mean daily temperature during the incubation period, temperature range during the first 24 hours and on day 4 post-infectious feed had a highly significant effect on the risk of infection. Deltamethrin exposure still significantly impaired survival of *kdr* homozygous mosquitoes, while mean daily temperature and relative humidity during the incubation period independently affected mosquito mortality. Significant differences in survival of resistant genotypes were detected, with the lowest survival recorded in mosquitoes with heterozygote *L1014S/L1014F* genotype.

**Conclusions:**

This study confirmed that the early part of sporogony is most affected by temperature fluctuations, while environmental factors affect mosquito survival. The impact of insecticide resistance on malaria infection and vector survival needs to be assessed separately for mosquitoes with different resistance mechanisms to fully understand its implications for currently available vector control tools and malaria transmission.

## Background

An increasing number of people in malaria endemic countries in sub-Saharan Africa have been protected by insecticide-treated nets (ITNs) or indoor residual spraying (IRS) [1]. Although the expansion of insecticide resistance in mosquitoes might endanger this progress [2, 3], there has not been a conclusive evidence of a complete failure of ITNs so far [4–8].

Longevity of vectors is one of the most important factors affecting malaria transmission [9, 10]. Vector mosquitoes must survive long enough to become infectious and transmit the disease to a new host. Environmental factors, especially ambient temperature and relative humidity affect *Anopheles* mosquitoes and parasite development. Temperature affects mosquito biting rates, blood meal digestion, duration of the gonotrophic cycle, fecundity, development of larval stages, and survival of larvae and adults [11]. It can also affect the immune system of mosquitoes [12–14] and consequently parasite development.

Duration of the sporogonic cycle is also temperature-dependent, with permissive range for *P. falciparum* sporogony being between 16 °C and 35 °C [15, 16]. Very high temperatures are lethal to parasites, while sporogony at low temperatures is lengthened to an extent that mosquitoes may not survive long enough to be able to transmit the parasites [17, 18]. The early part of sporogony is thought to be the most sensitive to temperature [12, 13, 18–24]. Ookinetes are the key transitional stage affecting the probability of vector infectivity, and also define thermal limits for parasite development. Once the parasites complete early sporogony and oocysts are formed, the effect of temperature is thought to be less damaging [19]. Both high and low temperatures have an effect, but the parasites are especially sensitive to high temperatures above 30 °C [19], and to temperature fluctuation immediately following the infectious blood meal [22].

Some studies have shown that the efficacy of insecticides against mosquitoes is to some extent temperature-dependent [25–28]. Furthermore, environmental factors such as temperature [25, 26, 29], larval food sources and availability of blood meals [30–34], infection by various parasites [35, 36], and insect microflora [37, 38] can all influence susceptibility to insecticides or expression of resistance.

When vectors are exposed to treated nets or sprayed surfaces, genetically resistant insects may survive doses that would have killed susceptible ones but might still be affected by the insecticide. Sub-lethal doses of pyrethroids were shown to reduce vector longevity and spontaneous flight activity, and to affect host seeking and probing responses [39, 40]. Such doses could potentially also affect mosquito physiology or trigger the immune response of mosquitoes [41]. Furthermore, sub-lethal doses of pyrethroids were shown to affect sporogonic development of *Plasmodium* parasites in laboratory conditions [39, 42–44], and also in the field [45]. The observed effects on parasite development could be caused by direct or indirect effect of insecticides, or through differential insecticidal killing of infected mosquitoes, as might be seen if infection restored phenotypic susceptibility in genotypically resistant mosquitoes.

Our aim in this study was to explore whether environmental variables such as temperature, together with the presence of the *kdr-L1014S* mutation, have an impact on survival probability and infection rates in wild *An. gambiae* (*s.s.*) exposed and unexposed to a pyrethroid insecticide.

## Methods

### Study area and participants

The study was conducted in Butemba, Kyankwanzi District, mid-western Uganda, between August 2013 and June 2014. Butemba is located at an altitude of 1000–1200 m above sea level in a moist savannah zone, with annual rainfall exceeding 1200 mm with two peaks (April-May and September-October). The area is highly endemic with two peaks of malaria transmission in May-July and October-December.

Forty-two gametocyte carriers were recruited among outpatients at Butemba Health Centre III. Volunteer patients who fulfilled the inclusion criteria (2 years or older, *P. falciparum*-positive with microscopically detectable gametocytes, no sign of severe illness, non-pregnant if adult female, and with a haemoglobin level of > 9.9 g/dl) were recruited. Gametocytes were counted against 200 leucocytes in thick blood smears. Density was calculated assuming a standard leukocyte count of 8000/μl of blood [46].

The experiments were carried out over three rounds (September-October 2013, November-December 2013, and May-June 2014).

### Mosquito collection and rearing

A non-air-conditioned field laboratory was established within the health centre for mosquito rearing and experiments. *Anopheles gambiae* (*s.l.*) larvae were collected from breeding sites in villages around the Health Centre and reared at the Health Centre at ambient temperature and humidity, in water from the breeding sites. The emerging adult mosquitoes were given 10% glucose solution until they were fed on infected blood.

### Experimental nets

Untreated polyester nets (Vestergaard Frandsen, Lausanne, Switzerland) were treated with a range of concentrations (2.5–16.7mg/m^2^) of deltamethrin (K-Othrine SC 10B G, concentration 9.7g/l; Bayer CropScience Ltd, Cambridge, United Kingdom) to ensure they were sub-lethal and constant across experiments. The doses were chosen in an attempt to mimic the concentrations found on nets as they get older in domestic use [47, 48], and were much lower than those used on LLINs. Sub-lethal doses were required to ensure sufficient survival of mosquitoes during the 7-day incubation period following an infectious blood meal and insecticide exposure to allow detection of any effects of deltamethrin, environmental variables and *kdr* resistance genotypes on parasite development.

### Procedures

Standard membrane feeding experiments were carried out as previously described [45]. Briefly, blood samples collected from gametocytaemic volunteers by venipuncture were transferred to pre-warmed membrane feeders (Hemotek Membrane Feeding System, Hemotek Ltd, Blackburn, UK) held at 37.5 °C. Approximately 40 female mosquitoes were placed in each paper cup and allowed to feed through an artificial Parafilm membrane for up to 2 h. Within 1–3 h following the feed, some of the blood-fed mosquitoes were exposed to a net treated with a sub-lethal dose of deltamethrin for 5 min using a wire ball frame, while others were exposed to an untreated net as control. After exposure, mosquitoes were kept in paper cups with access to 10% glucose solution. Seven days after infection, midguts of surviving females were dissected in 0.25% mercurochrome in phosphate buffered saline solution and examined for oocysts. Daily mortality of control and insecticide exposed mosquitoes was recorded.

### Mosquito processing

All mosquitoes were stored dry on silica gel in individual microtubes for molecular analysis. Real-time polymerase chain reaction (qPCR) using TaqMan assays was used for *Anopheles* sibling species identification [49], and for detection of *kdr-L1014F* or *kdr-L1014S* mutations [50]. A further assay to detect the presence of *G119S* mutation in the gene *ace-1* which encodes the acetylcholinesterase enzyme was also used [51].

### Temperature and relative humidity

Temperature and humidity were recorded every 30 min throughout the experiments, using EL-USB-2 data loggers (Lascar Electronics, Whiteparish, United Kingdom) placed next to the mosquito cages and pots in the laboratory.

### Statistical analysis

#### Software

Statistical analysis was carried out using Stata version 14 (StataCorp LP, College Station, Texas 77845, USA). Excel 2016 (Microsoft Corp) and Prism 7 (GraphPad Software Inc., La Jolla, CA 92037 USA) were used for data management and presentation of graphics.

#### Analysis of temperature and relative humidity variations between study rounds

One-way ANOVA with Tukey-Kramer’s *post-hoc* test [52] was used to compare temperature and relative humidity parameters between the three study rounds.

#### Effects of temperature and insecticide exposure on infection prevalence

Average daily temperatures, daily maximum and minimum temperatures, and daily temperature ranges (i.e. daily maximum minus minimum, indicating variation within a day) for each feed were obtained from the temperature records. Based on these, averages were also calculated for the period following the first 24 h post-infective blood meal until dissection day (i.e. day 7).

The effect on oocyst infection rates of temperature in the first 24 h post-feeding compared with subsequent days, together with deltamethrin exposure, was studied using mixed-effects logistic regression with backward elimination. Prevalence of oocyst infection among *An. gambiae* (*s.s.*) mosquitoes with *kdr-L1014S* homozygous genotype (692 mosquitoes) was studied as an outcome variable. Deltamethrin dosage group was entered as a categorical variable with three levels: control (untreated nets), low dose (2.5–5.0 mg/m^2^ deltamethrin) and high dose (10.0–16.7 mg/m^2^ deltamethrin). In addition, temperature-related variables were entered, including average daily temperature, daily maximum and minimum temperature, and daily temperature range on day 1 and during the period following the first 24 h post-feeding on infective blood meal until day 7. To account for the correlation of mosquitoes fed on the same blood sample within each experiment, gametocyte donor volunteers were included as a random (or group) variable.

#### Mosquito survival

Mosquito survival following the transmission experiments and insecticide exposure was studied among the *An. gambiae* (*s.s.*) with *kdr-L1014S* homozygous genotype, including the 692 mosquitoes which survived following the transmission experiments until day 7 and were successfully dissected, 53 mosquitoes which survived the period but were not successfully dissected, and 187 mosquitoes that died before day 7.

The influence of insecticide exposure and environmental variables on mosquito survival was studied using Kaplan-Meier survival curves and Log-Rank test.

The effect of temperature, relative humidity and deltamethrin exposure on mosquito survival through the seven days of incubation was studied using mixed-effects logistic regression with backward elimination. Mosquito mortality among *An. gambiae* (*s.s.*) mosquitoes with *kdr-L1014S* homozygous genotype was studied as an outcome variable. Gametocyte donor volunteers were included in the model as a random (or group) variable to account for the correlation of mosquitoes fed on the same blood sample within each experiment.

In addition, mosquito survival during the transmission experiments was studied among different *kdr* genotypes of *An. gambiae* (*s.s.*) present in the area, including 852 mosquitoes which survived following the transmission experiments until day 7, and 241 mosquitoes that died before day 7. Mortality of different *kdr* genotypes in *An. gambiae* (*s.s.*) mosquitoes used in transmission experiments was also compared using the Log-Rank test.

## Results

### PCR identification of *Anopheles gambiae* (*s.l.*) species

A total of 1196 *An. gambiae* (*s.l.*) were identified using PCR: 90.4% were found to be *An. gambiae* (*s.s.*)*,* and the rest were *An. arabiensis*. Further attempts to discriminate *An. coluzzii* were not made as this species was not expected to occur in the study area.

### Oocyst prevalence and intensity variations between study rounds

Significant variation in oocyst prevalence and oocyst intensity was observed between the rounds (Table 1). The lowest infection prevalence and intensity values were recorded in round 2 in all three insecticide dose categories. Within each round, both values were higher in mosquitoes that were not exposed to insecticides compared to those exposed.

**Table 1:** Oocyst prevalence and mean oocyst intensity (number of oocysts/midgut in infected mosquitoes) variation between the study rounds and doses of deltamethrin the mosquitoes were exposed to after infective feeds

Deltamethrin dose^a^	Study round^b^	Oocyst prevalence (%) (95% CI)	Comparison of prevalence between rounds	Mean number of oocysts/midgut (95% CI)	Analysis of variance
Control	1	92.7 (84.2–96.8)	*χ*^2^ = 94.14; *P* < 0.0001	12.45 (10.00–14.89)	*F*_(2, 252)_ = 12.32; *P <* 0.0001
	2	41.2 (23.3–61.8)		2.96 (2.33–3.59)	
	3	82.7 (67.3–91.8)		8.78 (6.75–10.80)	
Low dose	1	79.5 (44.8–94.9)	*χ*^2^ = 29.05 *P* = 0.0020	8.48 (5.95–11.01)	*F*_(2, 67)_ = 6.42; *P* = 0.0028
	2	25.8 (18.2–35.2)		1.00 (1.00–1.00)	
	3	81.6 (72.8–87.9)		5.03 (3.18–6.88)	
High dose	1	71.4 (52.9–84.7)	*χ*^2^ = 43.23 *P* = 0.0069	8.55 (6.58–10.51)	*F*_(2, 111)_ = 11.93; *P <* 0.0001
	2	23.7 (7.0–56.1)		2.17 (1.37–2.97)	
	3	60.0 (64.5–82.9)		4.86 (3.55–6.17)	

^a^Deltamethrin dose: Low dose, 2.5–5.0 mg/m^2^; high dose, 10.0–16.7 mg/m^2^

^b^Study rounds: 1, September-October 2013; 2, November-December 2013; 3, May-June 2014

### Temperature and relative humidity variations between study rounds

There was significant variation in mean daily temperature (T) (*F*_(2, 21)_ = 47.003, *P <* 0.0001) and maximum daily T (*F*_(2, 21)_ = 21.587, *P <* 0.0001) during the 7-day incubation period, and in daily T range during the same period (*F*_(2, 21)_ = 26.746, *P <* 0.0001). However, the mean minimum T during the incubation period was not significantly different between the three rounds (*F*_(2, 21)_ = 1.558, *P* = 0.234). Round 2 was on average the warmest, with the largest daily T variations. Rounds 1 and 3 were similar, but round 3 had slightly higher mean daily T during the incubation period (Table 2).

**Table 2:** Means of daily temperature (T), maximum and minimum temperature and daily temperature variation during the seven day incubation period, recorded during the three study rounds

	Mean daily T (°C)	Max T (°C)	Min T (°C)	T range (°C)
Round 1	25.2	27.8	23.4	4.3
Round 2	26.4	28.9	23.2	5.7
Round 3	25.4	28.0	23.4	4.6

Study rounds: 1, September-October 2013; 2, November-December 2013; 3, May-June 2014

There was also significant variation in all the relative humidity (RH) parameters: mean daily RH (*F*_(2, 21)_ = 216.85, *P <* 0.0001), minimum daily RH (*F*_(2, 21)_ = 97.334, *P <* 0.0001), maximum daily RH (*F*_(2, 21)_ = 132.1, *P <* 0.0001) and daily RH range (*F*_(2, 21)_ = 15.005, *P <* 0.0001) during the incubation period between the three rounds. The highest mean daily RH during the incubation period was measured in round 1, while RH in round 2 was the lowest.

### Temperature variations during first 24 hours post-infectious feed

**Fig. 1:**
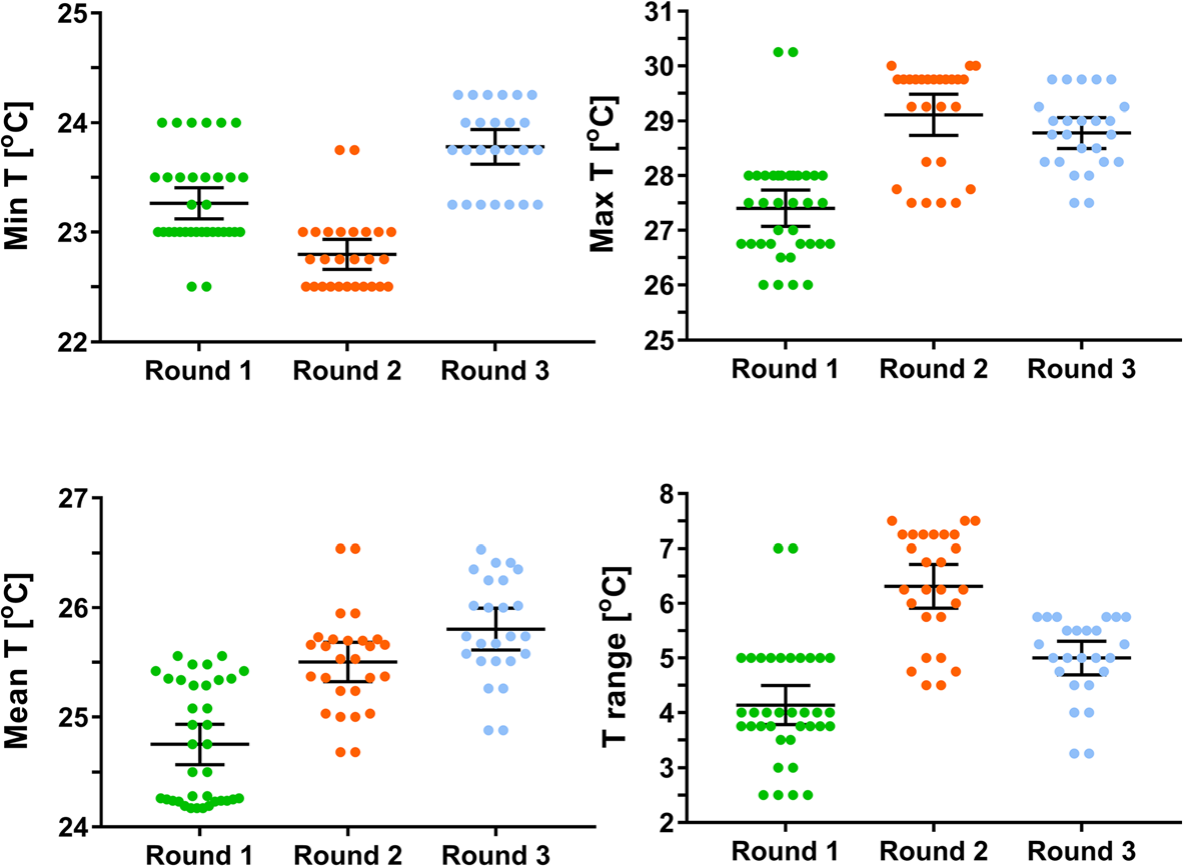
Temperature measurements during the first 24 h post-infectious feed recorded in each transmission experiment, in the three study rounds. There was significant variation in all temperature parameters during the first 24 h post-infectious feed between the three rounds: mean temperature (*F*_(2, 85)_ = 39.328, *P* < 0.0001), minimum temperature (*F*_(2, 85)_ = 41.749, *P* < 0.0001), maximum temperature (*F*_(2, 85)_ = 32.861, *P* < 0.0001) and the temperature range (*F*_(2, 85)_ = 36.57, *P* < 0.0001). Error bars show 95% confidence intervals of the means

Because the early part of sporogony, especially transition from zygotes into ookinetes and their passage through the midgut wall, is thought to be sensitive to temperature, the effects of temperature variables during the first 24 h post-infectious feed in each study round were studied in comparison with values in subsequent days (Fig. 1). There was significant variation in all the temperature parameters during the first 24 h post-infectious feed between the three rounds.

### Effect of temperature on oocyst prevalence

The effect of different temperature variables during the first 24 h post-infective feed and deltamethrin exposure on oocyst prevalence was investigated using mixed-effects logistic regression. A total of 692 *An. gambiae* (*s.s.*) homozygous for *kdr-L1014S* genotype from experiments that used blood samples from 42 gametocyte volunteers were included in the analysis. *Ace-1* mutation was not detected in any of the samples. The results showed that apart from the effect of deltamethrin on infection rates, temperature range during the first 24 h post-infectious feed (i.e. the difference between maximum and minimum temperature on day 1) which varied between 2.50 °C and 7.50 °C between different feeds, and temperature range on day 4 post-infectious feed which varied between 2.75 °C and 7.25 °C, had a highly significant effect on risk of infection (Table 3). The results indicate that an increase in temperature range was associated with lower infection, after controlling for the effects of insecticide exposure.

**Table 3:** Mixed-effects logistic regression analysis of *P. falciparum* oocyst prevalence rates

		OR	SE	*Z*	*P*	95% CI
Dose category	Control	1.000	–	–	–	–
	Low dose	0.423	0.139	-2.61	0.009	0.221–0.807
	High dose	0.190	0.048	-6.51	<0.0001	0.115–0.313
Temperature range day 1 (°C)		0.631	0.133	-2.18	0.029	0.417–0.954
Temperature range day 4 (°C)		0.606	0.137	-2.21	0.027	0.389–0.944
Variance of random intercept		2.092	0.729			1.057–4.143

Model *χ*^2^= 54.33, *df* = 3, *P* < 0.001; *n* = 692; number of groups (gametocyte donors) = 42

*Note*: The dependent variable is oocyst infection coded as 0 (negative) and 1 (positive)

*Abbreviations*: *OR* odds ratio, *SE* standard error, *95% CI* 95% confidence interval

### Mosquito survival in relation to temperature and humidity

Following the standard membrane feeds and insecticide exposure, fed mosquitoes were kept for seven days until dissection for the presence of oocysts. Mosquito mortality was recorded daily.

**Fig. 2:**
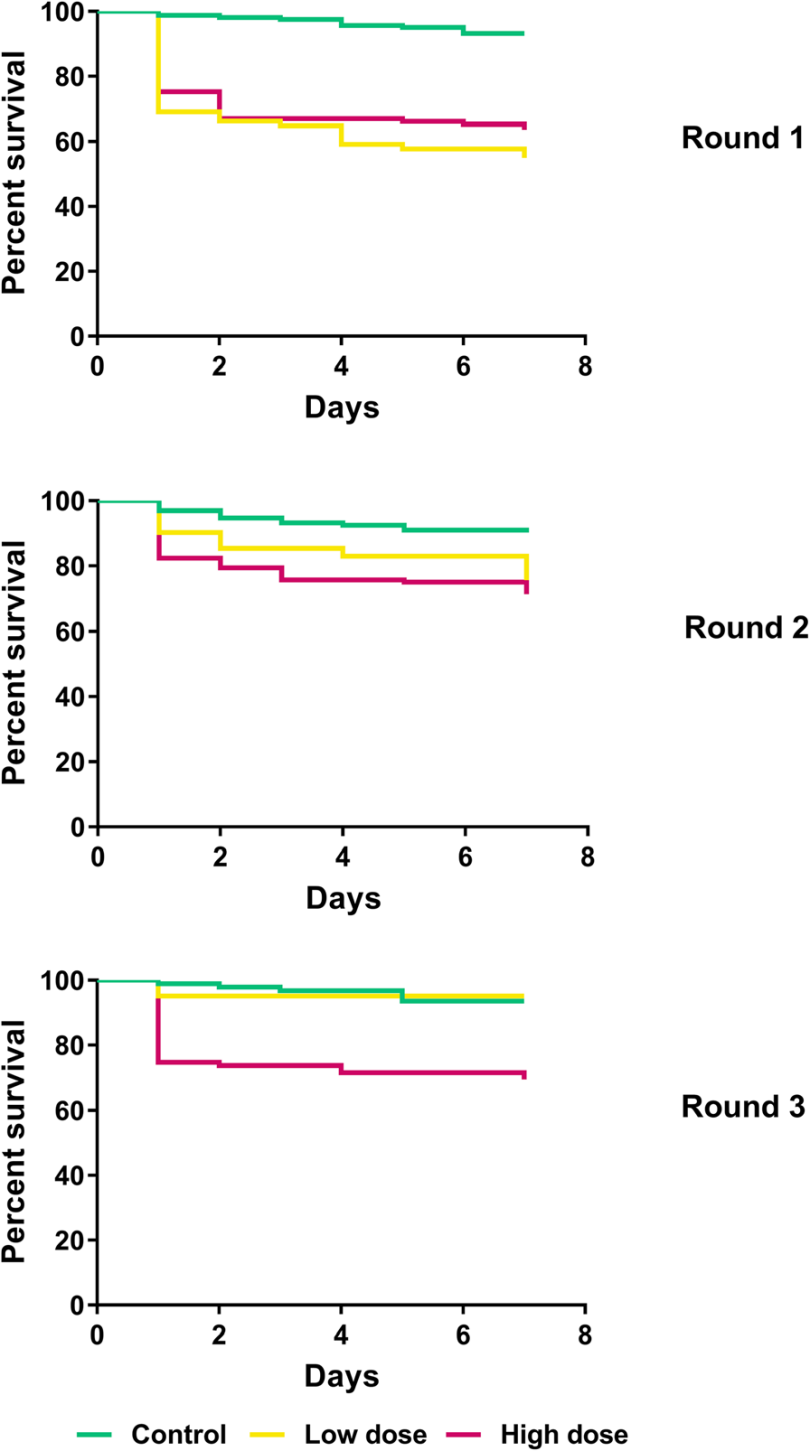
The Kaplan-Meier survival curves and estimates for mosquito survival in each of the three experimental rounds per each insecticide exposure dose. Survival distributions were significantly different between the insecticide exposure groups within each study round (Round 1 Log-Rank statistic, *χ*^2^ = 53.85, *df* = 2, *P* < 0.0001; Round 2 Log-Rank statistic, *χ*^2^ = 15.73, *df* = 2, *P* = 0.0004; Round 3 Log-Rank statistic, *χ*^2^ = 25.42, *df* = 2, *P* < 0.0001). Only *An. gambiae* (*s.s.*) mosquitoes homozygous for *kdr-L1014S* mutation were included in the analysis (control, untreated netting; low dose, 2.5–5.0 mg/m^2^ deltamethrin; high dose, 10.0–16.7 mg/m^2^ deltamethrin)

Figure 2 shows Kaplan-Meier survival curves for the three insecticide exposure groups within each study round. The survival curves showed the influence of insecticide exposure on mosquito survival, together with the influence of environmental variables. The survival distributions were significantly different between the insecticide exposure groups within each study round, showing that insecticide exposure impaired survival of *kdr* homozygous mosquitoes.

Mixed-effects regression analysis showed that apart from the effect of deltamethrin, average daily temperature and average daily relative humidity during the seven day incubation period had an independent and highly significant effect on mosquito mortality (Table 4).

**Table 4:** Mixed-effects logistic regression analysis of mortality rates of *An. gambiae* (*s.s.*) homozygous for *kdr-L1014S*

		OR	SE	*Z*	*P*	95% CI
Dose category	Control	1.000	–	–	–	–
	Low dose	5.144	1.588	5.31	<0.0001	2.809–9.422
	High dose	5.069	1.299	6.34	<0.0001	3.068–8.376
Average temperature (°C)		8.472	5.133	3.53	<0.0001	2.583–27.780
Average relative humidity (%)		1.248	0.073	3.80	<0.0001	1.113–1.399
Variance of random intercept		0.105	0.122			0.011–1.029

Model *χ*^2^ = 57.97, *df* = 3, *P* < 0.0001; *n* = 837; number of groups (gametocyte donors) = 42

*Note*: The dependent variable dead is mosquito death coded as 0 (alive) and 1 (dead)

*Abbreviations*: *OR* odds ratio, *SE* standard error, *95% CI* 95% confidence interval

**Fig. 3:**
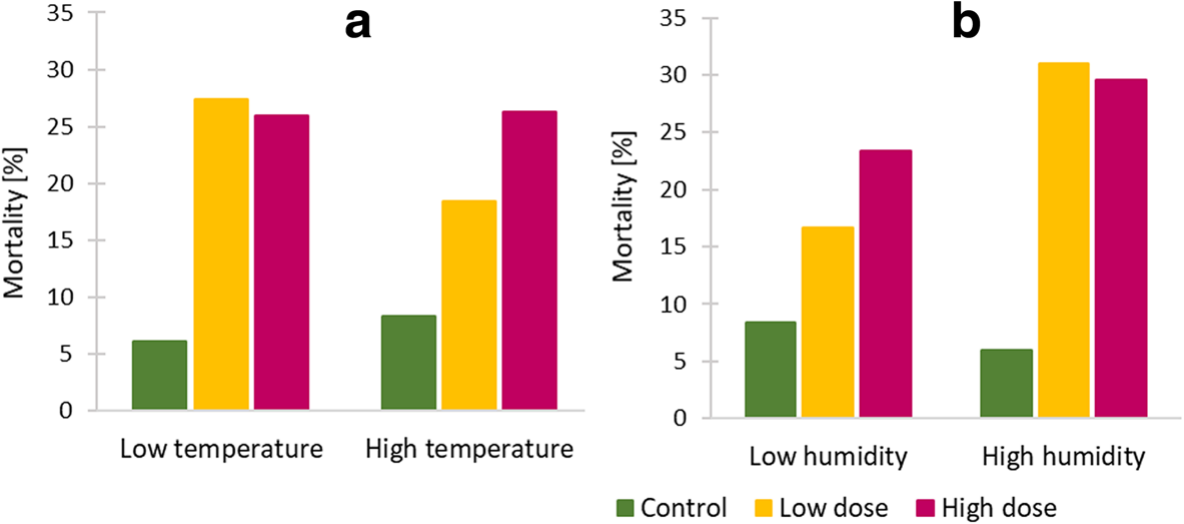
Effects of deltamethrin exposure on mosquito mortality in *kdr-L1014S* homozygous resistant *An. gambiae* (*s.s.*) mosquitoes. Mortality is shown (**a**) under low (< 25.3 °C) and high temperature (≥ 25.3 °C) conditions; (**b**) under low (< 69.7%) and high relative humidity (≥ 69.7%) conditions. Mosquitoes were exposed to control untreated nets, nets treated with low dose (2.5–5.0 mg/m^2^ deltamethrin) or high dose (10.0–16.7 mg/m^2^ deltamethrin) after feeding on blood samples from gametocytaemic volunteers. The median of ambient temperature recorded during the experiments (25.3 °C) and ambient relative humidity (69.7%) was used as a cut-off to plot mosquito mortality charts

Interactions of temperature, relative humidity and insecticide exposure and their effect on mosquito survival are shown in Fig. 3. Increased temperature and relative humidity resulted in increased mortality of deltamethrin-exposed mosquitoes compared with unexposed mosquitoes.

### Mosquito survival in relation to different *kdr* genotypes

**Fig. 4:**
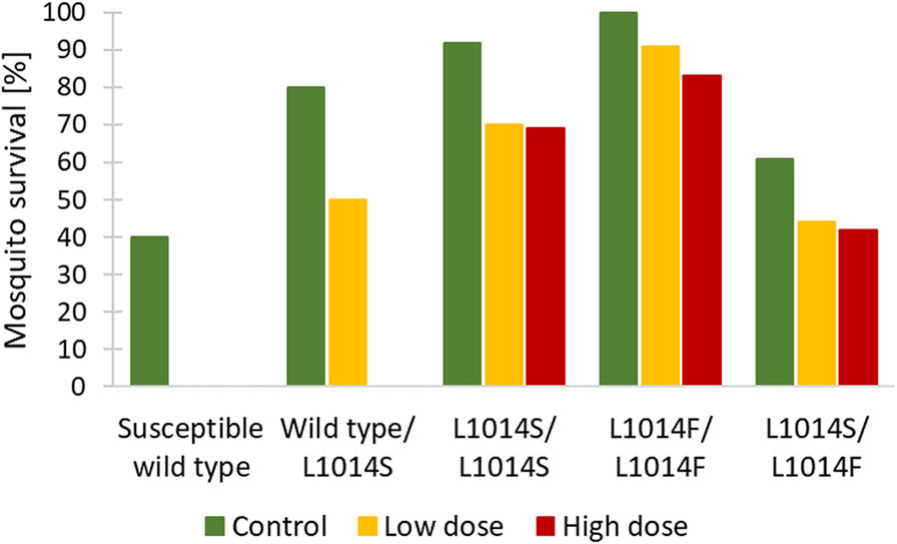
Survival of mosquitoes with different *kdr* genotypes following the membrane feeds and exposure to treated or untreated nets, was determined at the end of the seven day incubation period and compared per each exposure dose. Mosquitoes were exposed for 5 minutes using a wire ball frame to control untreated nets, nets treated with low dose (2.5–5.0 mg/m^2^ deltamethrin) or high dose (10.0–16.7 mg/m^2^ deltamethrin) after feeding on blood samples from gametocytaemic volunteers

Survival of mosquitoes with different *kdr* genotypes was compared following the membrane feeds and insecticide exposure (Fig. 4). No wild type susceptible mosquitoes or wild type/*L1014S* heterozygotes survived exposure to high doses of deltamethrin, although these two genotypes were present among the tested mosquitoes; low numbers of these samples did not allow for a more detailed analysis. Statistical tests of the effect of genotype on mortality did not provide a clear or consistent pattern among the three insecticide exposure groups. Survival of resistant homozygote *kdr-L1014S, kdr-L1014F* and heterozygote *L1014S/L1014F* genotypes was significantly different in control (Fisher’s exact test, *P* = 0.001), and high dose groups (Fisher’s exact test, *P* = 0.007), but not in a low dose group (Fisher’s exact test, *P* = 0.084). In all three insecticide exposure groups, a higher proportion of *L1014F/L1014F* genotype mosquitoes survived than of *L1014S/L1014S* mosquitoes, whereas the resistant heterozygote *L1014S/L1014F* genotype had the lowest survival of the three genotypes.

## Discussion

In order to determine whether environmental variables such as temperature and relative humidity, together with the presence of *kdr-L1014S* mutation, have an impact on survival probability and malaria infection, we compared daily survival and *Plasmodium* infection rates in wild insecticide-resistant *An. gambiae* (*s.s.*) fed on infective blood from gametocytaemic volunteers and exposed to untreated or deltamethrin-treated nets.

We have previously shown that average ambient temperature during the seven days of incubation, together with insecticide exposure, had a highly significant effect on the risk of infection in mosquitoes and on the parasite load [45]. In the present study, we wanted to further explore any possible effects of different environmental variables on parasite development and vector survival, in the presence or absence of insecticide exposure and in different *kdr* genotypes. Apart from insecticide dose, temperature range on day 1 (i.e. within the first 24 hours) and on day 4 post-infective blood meal had significant effects on parasite development.

The period of the first 24 hours following the infective feed is the time of blood meal digestion and early sporogony, with ookinete densities reaching peak numbers [20] while the peritrophic matrix, which the ookinetes must traverse, reaches its maximal thickness [53]. This part of sporogony is particularly sensitive to both temperature [12, 13, 18–24] and exposure of infected mosquitoes to sub-lethal doses of pyrethroids [39]. Blood meal digestion in mosquitoes is temperature-dependent [54], while the speed of digestion also affects the sporogony, especially formation of ookinetes and their migration through the peritrophic matrix and the midgut wall [55]. During study round 2, temperature variations in the first 24 hours post-feed were significantly larger than in rounds 1 or 3, whereas parasite prevalence and intensity of infection were significantly lower, regardless of insecticide exposure.

Young oocysts can be seen from day 2 post-infection [56]. During this period, mitotic divisions start taking place, forming a multinucleate oocyst, and circumsporozoite protein (CSP) must be produced for formation and budding of the sporozoites [57, 58]. Although it is possible that some of the processes taking place during sporozoite development in the oocysts are temperature-sensitive, previous studies show that oocysts, once formed, are no longer sensitive to changes in ambient temperature [19]. A recent study shows that not all mosquitoes are equally infectious as those with lower sporozoite burdens have a lower chance of successfully spreading the infection [59]. Further work will be required to assess the impact of sub-lethal doses of pyrethroids, of environmental variables and insecticide resistance on sporozoite development and infectivity.

Exposure to insecticides, ambient temperature and relative humidity, malaria infection and insecticide resistance all interact in nature and can affect vector competence in differing ways, but their combined effect on mosquito survival is not well understood. Increase in environmental temperature has been shown to be associated with reduced adult survival [60, 61]. Temperature also affects the extent to which insecticides kill mosquitoes [25, 26], possibly because mosquito immune responses [12], nervous-system sensitivity [62], and metabolic activity [63] are all temperature-dependent. Apart from its effect on mosquito survival in combination with ambient temperature [11], humidity was shown to have a strong impact on insecticide resistance phenotype [64].

Insecticide resistance mechanisms can also exert a wide range of effects on vector longevity, competence and behavior and could in principle affect malaria transmission in either a positive or negative manner [65]. Moreover, different resistance alleles can interact to influence the fitness of mosquitoes [66]. Extensive comparison of survival between different *kdr* genotypes and the wild type was not possible due to low numbers of mosquitoes with a wild type allele. However, survival over the seven day incubation period (during which the oocysts developed) of mosquitoes with resistant genotypes (i.e. with at least one resistant allele, *L1014S* or *L1014F*) was higher than survival of the wild susceptible type. There were also significant differences in survival over the incubation period of resistant *L1014S/L1014S*, *L1014F/L1014F* and *L1014S/L1014F* genotypes in control and high dose groups; in both instances, survival was the lowest in mosquitoes with *L1014S/L1014F* genotype, which could be due to combination of resistant alleles exerting a fitness cost on mosquitoes. Several properties of infected blood can impair mosquito fitness, even in the absence of actual mosquito infection, while survival of infected mosquitoes is also affected by environmental stress [67]. Furthermore, survival of uninfected *kdr* resistant mosquitoes was shown to be higher than that of the susceptible strain, while their survival was similar when exposed to *P. falciparum* infection [68].

## Conclusions

This study allowed us to examine the relationships between environmental variables and insecticide exposure on survival probability and infection rates in wild *An. gambiae* (*s.s.*) in the presence of *kdr-L1014S* mutation. As previously observed, early sporogony was most sensitive to temperature, especially to temperature variation, regardless of the insecticide exposure. We also show that temperature and relative humidity, together with insecticide exposure, impact mosquito survival following infected feeds. From a vector control perspective, it was encouraging to find that deltamethrin exposure still significantly impaired survival of *kdr* homozygous mosquitoes. The impact of insecticide resistance on malaria infection and vector survival needs to be assessed separately for mosquitoes carrying target site or metabolic resistance mechanisms before we will be able to fully understand the impact of resistance on currently available vector control tools and on malaria transmission.
